# Graph analysis of verbal fluency test discriminate between patients with Alzheimer's disease, mild cognitive impairment and normal elderly controls

**DOI:** 10.3389/fnagi.2014.00185

**Published:** 2014-07-29

**Authors:** Laiss Bertola, Natália B. Mota, Mauro Copelli, Thiago Rivero, Breno Satler Diniz, Marco A. Romano-Silva, Sidarta Ribeiro, Leandro F. Malloy-Diniz

**Affiliations:** ^1^Laboratory of Clinical Neuroscience Investigations, Federal University of Minas GeraisBelo Horizonte, Brazil; ^2^Brain Institute, Federal University of Rio Grande do NorteNatal, Brazil; ^3^Physics Department, Federal University of PernambucoRecife, Brazil; ^4^Mental Health Department, Faculty of Medicine, Federal University of Minas GeraisBelo Horizonte, Brazil; ^5^Faculty of Medicine, National Institute of Science and Technology – Molecular Medicine, Federal University of Minas GeraisBelo Horizonte, Brazil

**Keywords:** semantic verbal fluency, graph analysis, elderly, Alzheimer's disease, mild cognitive impairment

## Abstract

Verbal fluency is the ability to produce a satisfying sequence of spoken words during a given time interval. The core of verbal fluency lies in the capacity to manage the executive aspects of language. The standard scores of the semantic verbal fluency test are broadly used in the neuropsychological assessment of the elderly, and different analytical methods are likely to extract even more information from the data generated in this test. Graph theory, a mathematical approach to analyze relations between items, represents a promising tool to understand a variety of neuropsychological states. This study reports a graph analysis of data generated by the semantic verbal fluency test by cognitively healthy elderly (NC), patients with Mild Cognitive Impairment—subtypes amnestic (aMCI) and amnestic multiple domain (a+mdMCI)—and patients with Alzheimer's disease (AD). Sequences of words were represented as a speech graph in which every word corresponded to a node and temporal links between words were represented by directed edges. To characterize the structure of the data we calculated 13 speech graph attributes (SGA). The individuals were compared when divided in three (NC—MCI—AD) and four (NC—aMCI—a+mdMCI—AD) groups. When the three groups were compared, significant differences were found in the standard measure of correct words produced, and three SGA: diameter, average shortest path, and network density. SGA sorted the elderly groups with good specificity and sensitivity. When the four groups were compared, the groups differed significantly in network density, except between the two MCI subtypes and NC and aMCI. The diameter of the network and the average shortest path were significantly different between the NC and AD, and between aMCI and AD. SGA sorted the elderly in their groups with good specificity and sensitivity, performing better than the standard score of the task. These findings provide support for a new methodological frame to assess the strength of semantic memory through the verbal fluency task, with potential to amplify the predictive power of this test. Graph analysis is likely to become clinically relevant in neurology and psychiatry, and may be particularly useful for the differential diagnosis of the elderly.

## Introduction

Language and semantic memory tend to remain stable across the human lifespan in contrast to other cognitive domains, like episodic memory and attention, which usually decline after the 5th decade (Craik and Bialystok, 2006). They are also usually spared in the initial stages of neurodegenerative disorders, such as Alzheimer's disease (AD), though we can still observe milder deficits, e.g., anomia or reduced semantic verbal fluency, which can be identified in a comprehensive neuropsychological evaluation (Henry et al., 2004; Garrard et al., 2005; Nutter-Upham et al., 2008; Taler and Phillips, 2008).

Verbal fluency is the ability to produce a satisfying sequence of spoken words during a given time interval (Nickles, 2001). Verbal fluency tests are experimentally designed to assess this ability through the production of words starting with a specific letter (Phonemic Verbal Fluency) or belonging to a category of knowledge (Semantic Verbal Fluency). Semantic verbal fluency is one of the most commonly used tasks to evaluate language and semantic memory skills in older adults. This task depends on the preservation of language (e.g., words can be spoken correctly during the task), though it is significantly influenced by semantic memory (e.g., the knowledge of the category asked must be intact) and executive function (e.g., the ability to search the asked knowledge) domains (Adlam et al., 2006; Unsworth et al., 2011). This task often activates the temporal lobe, a region broadly related to conceptualization, general information and knowledge about names (Patterson et al., 2007). Semantic verbal fluency contributes to predict future cognitive and functional impairments in the elderly (Salmon et al., 2002; Amieva et al., 2005; Hodges et al., 2006; Aretouli et al., 2011), and predict the progression from MCI to AD (Saxton et al., 2004).

Despite being widely used for neuropsychological assessment in the elderly, the standard measure of the verbal fluency test is restricted to the total of correct words produced in the task (Lezak et al., 2004; Strauss et al., 2006), and does not take into account other clinically-relevant information that may be contained in the patient's specific performance. This task requires the production of words belonging to a specific category, and each subject produced the words following an order of exemplars during the 1-min task. This order of words produced allows the construction of a network based on the temporal link between the words. These temporal links may inform that words produced in a specific temporal sequence are probably conceptually related, as suggested by the semantic association models (McClelland and Rogers, 2003; Griffiths et al., 2007).

Goni et al. (2010) constructed a semantic network using the verbal fluency task applied to an adult sample, and represented the semantic memory as a graph ruled by conceptual constraints. A normal semantic verbal fluency network is represented by a directed graph with only one occurrence for each word. Lerner et al. (2009) investigated the network properties of subjects with MCI and AD, and found that the path lengths of the network decline while the clustering coefficient increases in the MCI and AD subjects compared to healthy elderly controls. These results showed that the normal characteristics of the semantic verbal network are significantly changed in the continuum from normal aging to AD.

The analysis of network properties helps understanding the dynamics and organization of the cognitive and behavioral processes. A graph represents a network with nodes linked by edges (Mota et al., 2012). Formally, a graph is a mathematical representation of a network G = (N, E), with N = {w_1_, w_2_, … w_n_} a set of nodes and E = {(w_i_, w_j_)} a set of edges or links between words w_i_ in N and w_j_ in N. The interpretation of the meaning of a graph depends on what is being represented (Butts, 2009; Mota et al., 2012). We carried out an analysis of the network properties of the semantic verbal fluency of subjects with MCI or AD. We hypothesize that the analysis of the semantic verbal fluency network properties can help to better discriminate between older adults with normal cognitive performance, mild cognitive impairment or Alzheimer's disease. This approach had been used with success to identify patients with schizophrenia and bipolar disorder (Mota et al., 2012, 2014).

## Materials and methods

### Subjects

One hundred older adults were included in this study. All subjects were assessed in the Centro de Referência à Saúde do Idoso Jenny de Andrade Faria, Clinical Hospital, Federal University of Minas Gerais. All the participants underwent a comprehensive clinical and neuropsychological assessment. The neuropsychological protocol included the following tests: Mini Mental State Exam, Frontal Assessment Battery, Category Verbal Fluency of Animals and Fruits, Letter Fluency of S, Digit Span, Stick Design Test, Clock Drawing Test, Rey Auditory Verbal Learning Test, Naming Test (TN-LIN), and Token Test. This protocol has been validated for the neuropsychological assessment of older adults with low educational status (de Paula et al., 2013). After the clinical and neuropsychological assessment, and adjudication meeting was held and the final diagnosis was reached by consensus. The AD diagnosis was based on the proposed criteria of McKhann et al. (1984) and the patient should present general and worsening cognitive impairment, in two or more cognitive domains, and functional impairment in the daily living activities. The MCI diagnosis followed the criteria proposed by Winblad et al. (2004), were the older adult presents cognitive decline in one or more cognitive domains but is preserved in basic and instrumental daily living activities or presents a minimal impairment. The MCI subgroup division considered the amnestic MCI (aMCI) classification for participants that only present memory impairment, and amnestic multiple-domain MCI (a+mdMCI) for participants that present impairment in memory and other cognitive domain, though fulfilling all the MCI criteria established by Winblad et al. (2004).

The project was approved by the Research Ethics Committee of the Federal University of Minas Gerais (COEP-334/06). The subjects were divided into four groups: (1) normal cognitive performance (NC), *n* = 25; (2) amnestic single-domain MCI (aMCI), *n* = 25; (3) amnestic multiple-domain MCI (a+mdMCI), *n* = 25; (4) AD, *n* = 25.

### Verbal fluency test

The participants performed the Semantic Verbal Fluency test, category of animals, for which they were asked to produce the maximum names of animals within 60 s; explicit/implicit instructions were given to avoid repetitions. All the words were recorded, including repetitions and errors. The scoring procedure included: total of words produced, total of correct words, total of errors, total of repetitions, and the fraction of repetitions according to the total of words produced by each participant. The scores in this task were not taken into account in the diagnosis adjudication of each participant.

### Statistical analysis

The study design involved two stages of analysis, considering three (NC, MCI, AD) or four groups (NC, aMCI, a+mdMCI, AD), and the same statistical analysis and graph measures were performed for comparing the three or four groups. The MCI group comprised both the aMCI and the a+mdMCI groups.

We performed the Shapiro-Wilk test of normality of the sample, and since the majority of the variables did not fit the assumption of normality, we used the Kruskal-Wallis test of differences between several independent groups and the Wilcoxon Rank sum test for two independent samples. Bonferroni correction was applied to all analyses.

Group sorting was implemented with a Naïve Bayes classifier, which shows superior performance with small samples (Singh and Provan, 1995; Kotsiantis, 2007). The choice of attributes for the classifier was based on significant correlations of the attributes with established clinical measures of differential diagnosis (global cognitive status and daily living functionality). Sensitivity, specificity and the area under the receiver operating characteristic curve (AUC) were used to estimate classification quality, which was considered excellent when AUC was higher than 0.8, good when AUC ranged from 0.6 to 0.8, and poor (not above the chance), when AUC was smaller than 0.6.

### Graph measures

**Figure 1:**
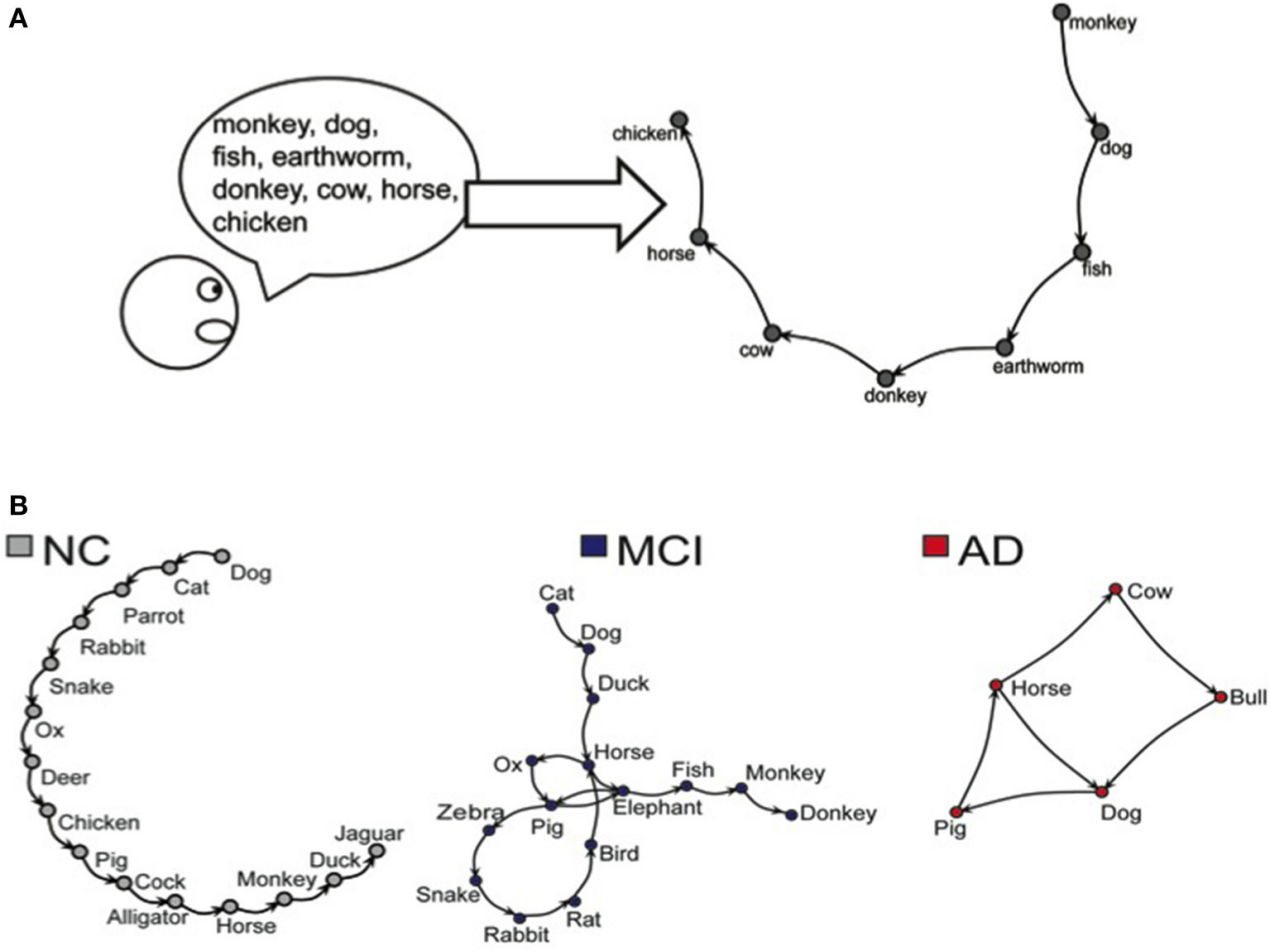
**(A)** Representation of the word sequence produced on the Semantic Verbal Fluency task. **(B**) Representations of networks generated by NC, MCI, and AD subjects during the Semantic Verbal Fluency task.

The word sequence produced on the Semantic Verbal Fluency test was represented as a speech graph, using the software *SpeechGraphs* (Mota et al., 2014). The program represents a text (in this case, the sequence of words produced by the verbal fluency test) as a graph, representing every word as a node, and the temporal link between words as an edge (Figure 1).

We then calculated word count (WC) and 13 additional Speech Graph Attributes (SGA) comprising general attributes: total of nodes (N) and edges (E); connected components: the largest strongly connected component (LSC); recurrence attributes: repeated (RE) and parallel edges (PE), cycles of one (L1), two (L2), or 3 nodes (L3); global attributes: average total degree (ATD), density, diameter, average shortest path (ASP) and clustering coefficient (CC) (for more detailed information see Supplementary Table and Figure on Supplementary Material).

Given the task instructions, we expected the subjects to produce a linear network, i.e., a sequence in which each correct word was followed by a different correct word, without repetitions. A correct performance in this test should yield graphs with identical number of nodes (N) and words (WC), N-1 edges, no recurrence (i.e., without parallel edges, repeated edges or loops), and zero strongly connected components (LSC). In addition, the average total degree (ATD) should be close to 2, with a very small density, very low clustering coefficient (CC), and large distances (diameter should be equal to E).

## Results

Table 1 shows data for socio-demographic data, Mini Mental State Exam (MMSE), total number of produced words in the verbal fluency test, total number of correct words produced, total number of repetitions performed during the task, the percentage of repetitions performed according to the total of produced words, and the errors produced.

**Table 1:**
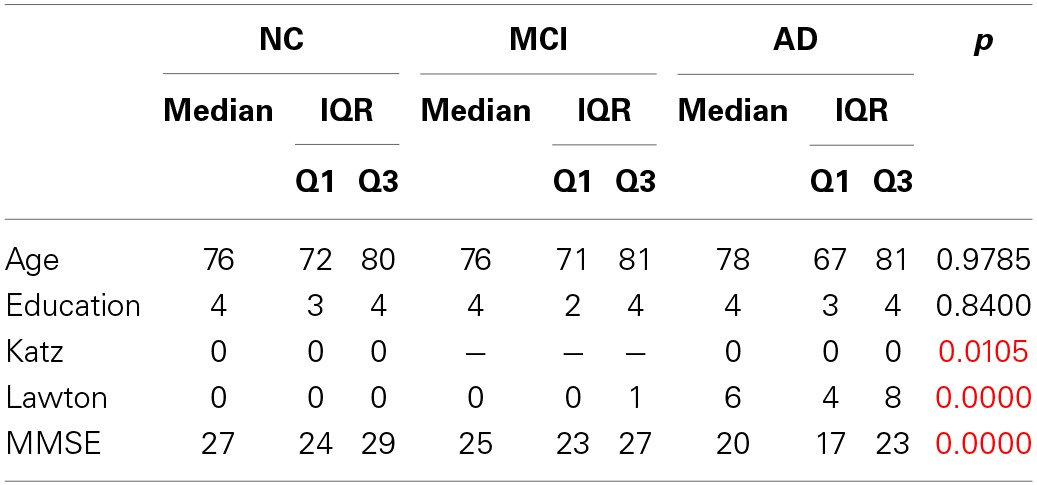
**Socio-demographic data, verbal fluency and Speech Graph Attributes of NC, MCI, and AD groups, with Bonferroni-corrected significant differences across groups established by the Kruskal-Wallis comparison**.

*Katz, Katz Index; Lawton, Lawton Index; MMSE, Mini Mental State Exam; IQR, Interquartile Range; Q1, 1th Quartile; Q3, 3trd Quartile. Red values have significance p = 0.0167*.

The groups did not differ in age and education, and only the control group had a significant difference in gender distributions (*X*^2^ = 6.76, *df* = 2, *p* = 0.009) (Table 1). The results of the groups' comparison on the daily living activities, the global cognitive status are also reported on Table 1. Verbal fluency measures and the Speech Graph Attributes are reported on Table 2.

**Table 2:**
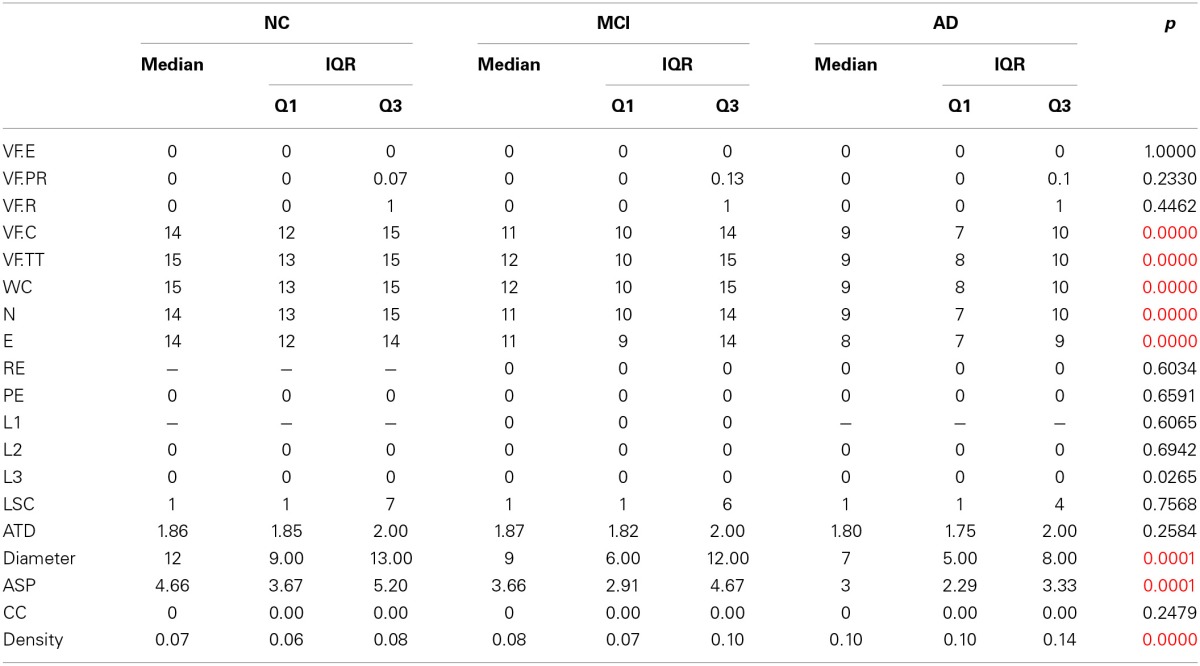
**Verbal fluency and Speech Graph Attributes of NC, MCI, and AD groups, with Bonferroni-corrected significant differences across groups established by the Kruskal-Wallis comparison**.

VF.E, errors; VF.PR, percentage of repetitions; VF.R, repetitions; VF.C, corrects words; VF.TT, total of words; WC, word count; N, nodes; E, edges; RE, repeated; PE, parallel edges; L1, L2, L3, cycles of one, two or 3 nodes; LSC, largest strongly connected component; ATD, average total degree; ASP, average shortest path; CC, clustering coefficient. Red values have significance p = 0.0167.

Despite the lower number of correct words produced by the NC group, it is similar to those observed to Brazilian normative data (Brucki et al., 1997). Moreover, the scores on the verbal fluency test were not taken into account for participant classification into the diagnostic groups.

The groups significantly differed in the performance on ADLs, in general cognitive status, number of correct words and total words produced, and in the Speech Graph measures of word count, nodes, edges, loops of 3 nodes, diameter, average short path and density. As expected, the NC group performed better at ADLs, had higher scores on the MMSE, produced more nodes, a network with larger diameter and less dense, when compared with the MCI and AD groups. The MCI group showed an intermediate performance between NC and AD groups in all measures.

**Figure 2:**
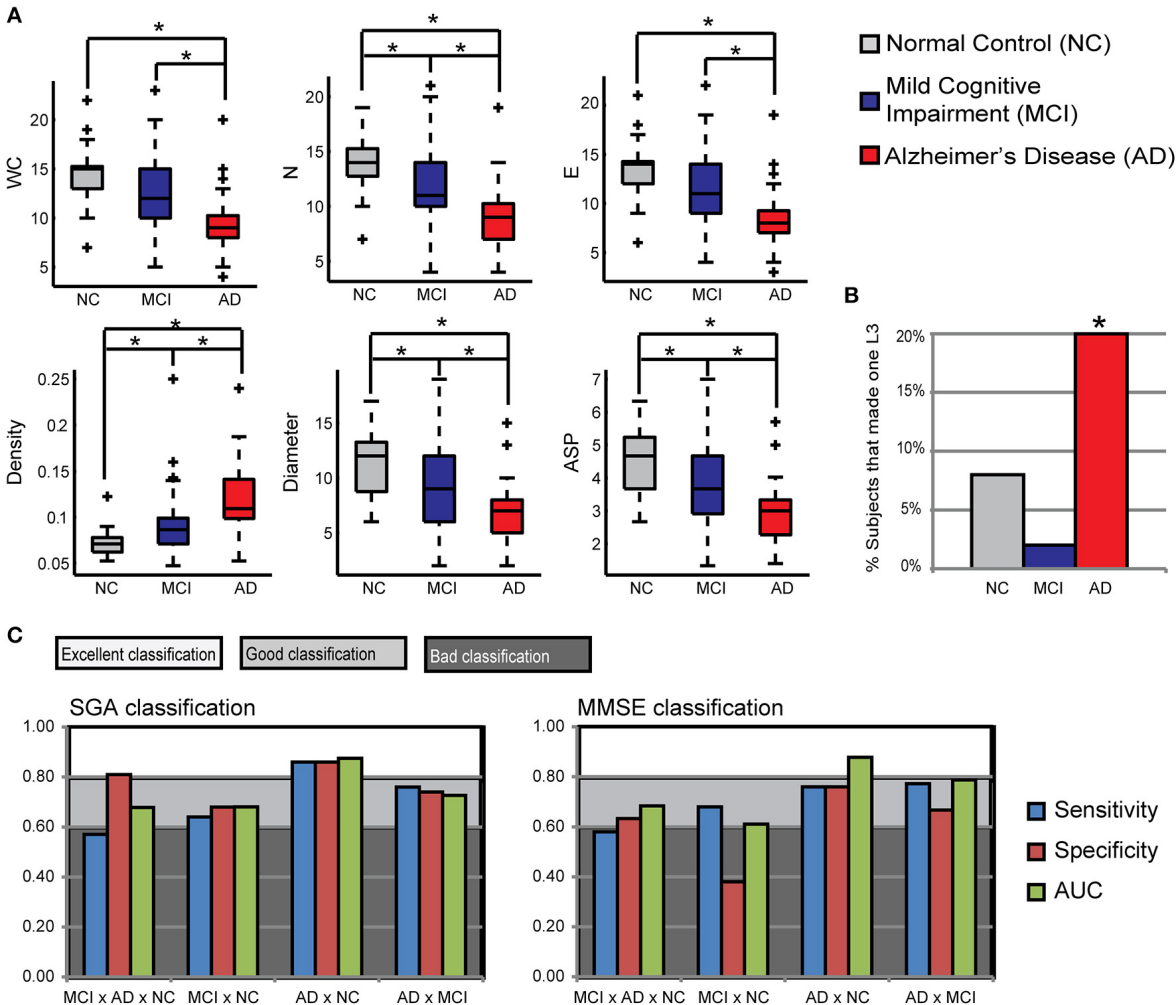
**Speech Graph Attributes (SGA) differentiates psychopathological groups. (A)** SGA boxplots with significant differences among Alzheimer Disorder (AD), Moderate Cognitive Impairment (MCI) and control groups (*N* = 25 on AD and C group, *N* = 50 on MCI group; Kruskal-Wallis test followed by two-sided Wilcoxon Rank-sum test with Bonferroni correction with alpha = 0.0167). **(B)** Percentage of subjects in each group that made one L3 on the verbal fluency test. AD subjects showed more L3 than MCI subjects (Wilcoxon Rank-sum test with Bonferroni correction with alpha = 0.0167, *p* = 0.0090). **(C)** Rating quality measured by AUC, sensitivity and specificity, using MMSE or SGA correlated with clinical symptoms measured with MMSE and Lawton scales (Table 3) (attributes: WC, N, E, Density, Diameter, and ASP). Notice that SGA was more specific than MMSE on triple group sorting, and on MCI diagnosis against the control group. ^*^p = 0.0167.

Table 3 and Figure 2A show pairwise comparisons of the 3 diagnosis groups. Statistical significance was set at *p* < 0.0167, after Bonferroni correction for multiple comparisons.

**Table 3:**
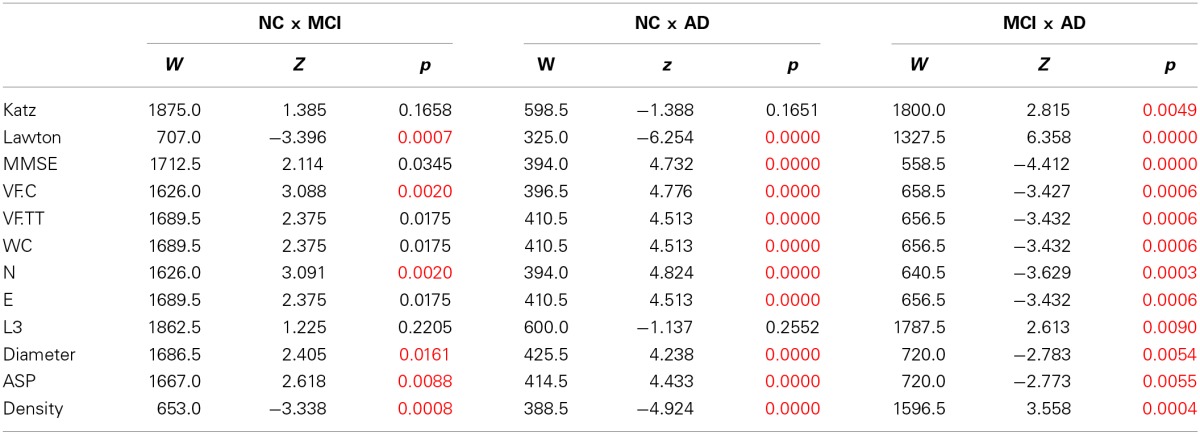
**Pairwise group comparison with Bonferroni-corrected significant differences between groups established by Wilcoxon Ranksum test**.

*W, Wilcoxon Ranksum; z, = z score. Red values have significance p = 0.0167*.

The comparison of the variables between NC and MCI groups demonstrate that the groups differ in the index of instrumental daily living activities, in the number of correct words produced, number of nodes, diameter, average short path and density of the network. The NC produced less dense graphs with more nodes, and larger Diameter and ASP than the MCI and AD. Furthermore, NC made more edges, total words produced, and had a better general cognitive status than the AD group. The MCI and AD groups differ in all measures, demonstrating that a change in the general cognitive status, functionality, verbal fluency measures and the speech graph attributes (WC, N, E, L3, Diameter, ASP, and Density) (Figure 2B) almost follow a continuous modification as the diagnosis impairs.

Table 4 shows the Spearman correlations between the SGA and the clinical measures of differential diagnosis (global cognitive status—MMSE—and daily living functionality—Katz and Lawton Index). The significance level was established in *p* = 0.0012 after a Bonferroni correction for 42 comparisons.

**Table 4:**
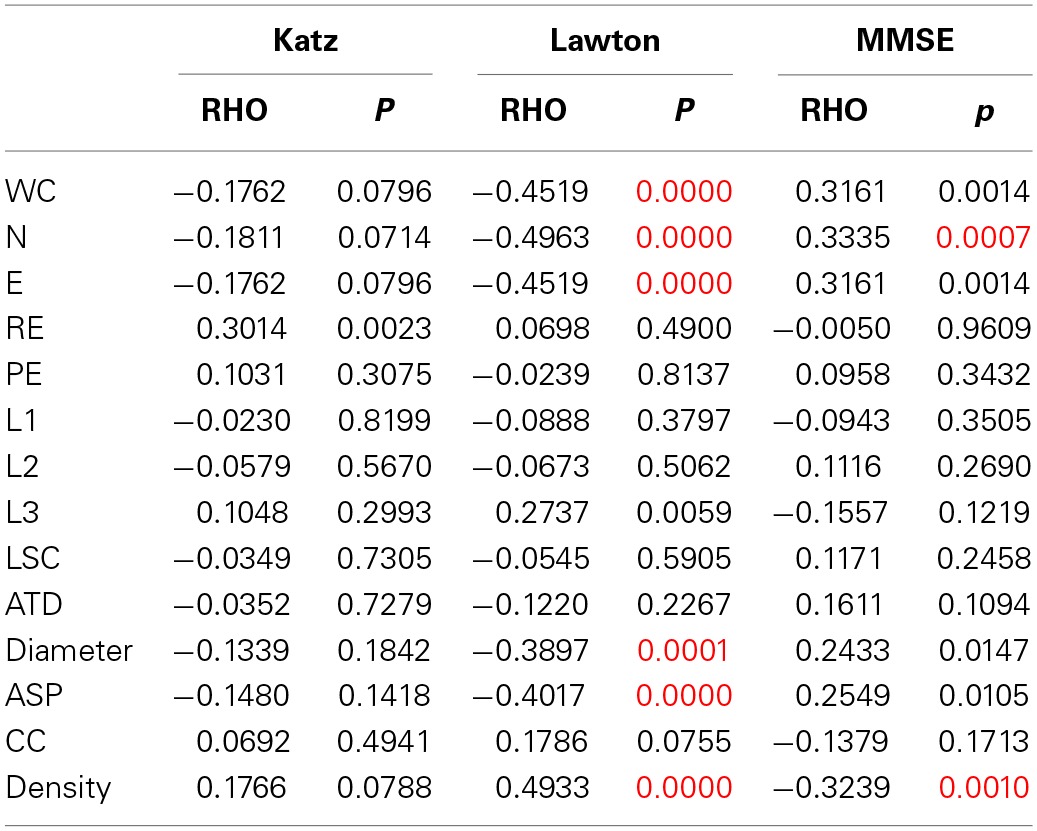
**Spearman correlation (RHO and *p*-values) between SGA scores and the Katz, Lawton or MMSE scores**.

*Red values have significance p = 0.0012*.

We found significant correlations between the MMSE and the SGA of Nodes and Density, indicating that the more cognitively preserved elderly produced a larger number of unique nodes, and networks with a smaller density than cognitively impaired subjects. The correlation between the attributes and the Lawton Index of instrumental daily living activities revealed that the more functionally dependent were the elderly, the less words, nodes and edges they produced, showing networks with a smaller diameter and average short path, but a higher density. These results indicate that functional autonomy correlate more with SGA than with the general cognitive status.

The Naïve Bayes classifier results (Figure 2C) show that a selection of SGA correlated with functional and cognitive impairment measured by other instruments, provided good to excellent classification power, being similar to the MMSE classification power, or even better for the distinction between the NC and MCI groups. When the SGA were associated to the Lawton Index or the MMSE, the power of classification increased; a combination of the 3 measurements provided maximal classification quality (Table 5). Overall, the combination of graph measures and functional dependence yielded very accurate differential classification of the AD (1.00) and MCI (0.78) against the NC group, and between the MCI and AD (0.84).

**Table 5:** **Rating quality measured by AUC, using SGA (attributes: WC, N, E, Density, Diameter, and ASP) correlated with clinical symptoms measured with MMSE and Lawton scales, in addition with Lawton, MMSE or both, classifying AD and MCI from NC, AD from MCI, and also classifying subtypes of MCI (aMCI or a+mdMCI) from NC or AD, or from each other**.

		**SGA**	**SGA+MMSE**	**SGA+ Lawton**	**SGA+MMSE+Lawton**	**MMSE**	**Lawton**
NC x	MCI	0.681	0.716	0.780	0.793	0.638	0.649
	aMCI	0.619	0.618	0.710	0.714	0.586	0.612
	a+mdMCI	0.710	0.738	0.803	0.822	0.694	0.746
	AD	0.875	0.886	1.000	1.000	0.888	1.000
aMCI x	a+mdMCI	0.486	0.470	0.472	0.483	0.631	0.494
	AD	0.767	0.824	0.856	0.856	0.854	0.957
a+mdMCI x	AD	0.652	0.717	0.814	0.811	0.772	0.959
MCI x	AD	0.727	0.793	0.849	0.858	0.813	0.958

The additional description of the socio-demographic data, Mini Mental State Exam (MMSE), verbal fluency measures of the two subgroups of MCI are reported on Table 6, and also the results of the four groups' comparison on the sociodemographic variables. Table 7 shows the four group comparison on the verbal fluency and Speech Graph Attributes.

**Table 6:**
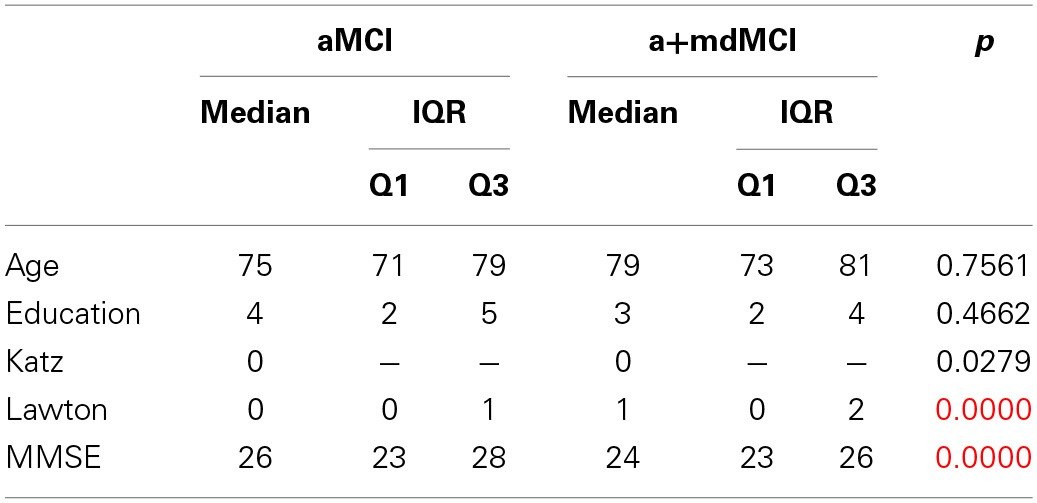
**Additional description of socio-demographic data for the MCI subtypes, and the four groups comparison**.

p^*^ group comparison (NC; aMCI; a+mdMCI; AD). Red values have significance p = 0.0083.

**Table 7:**
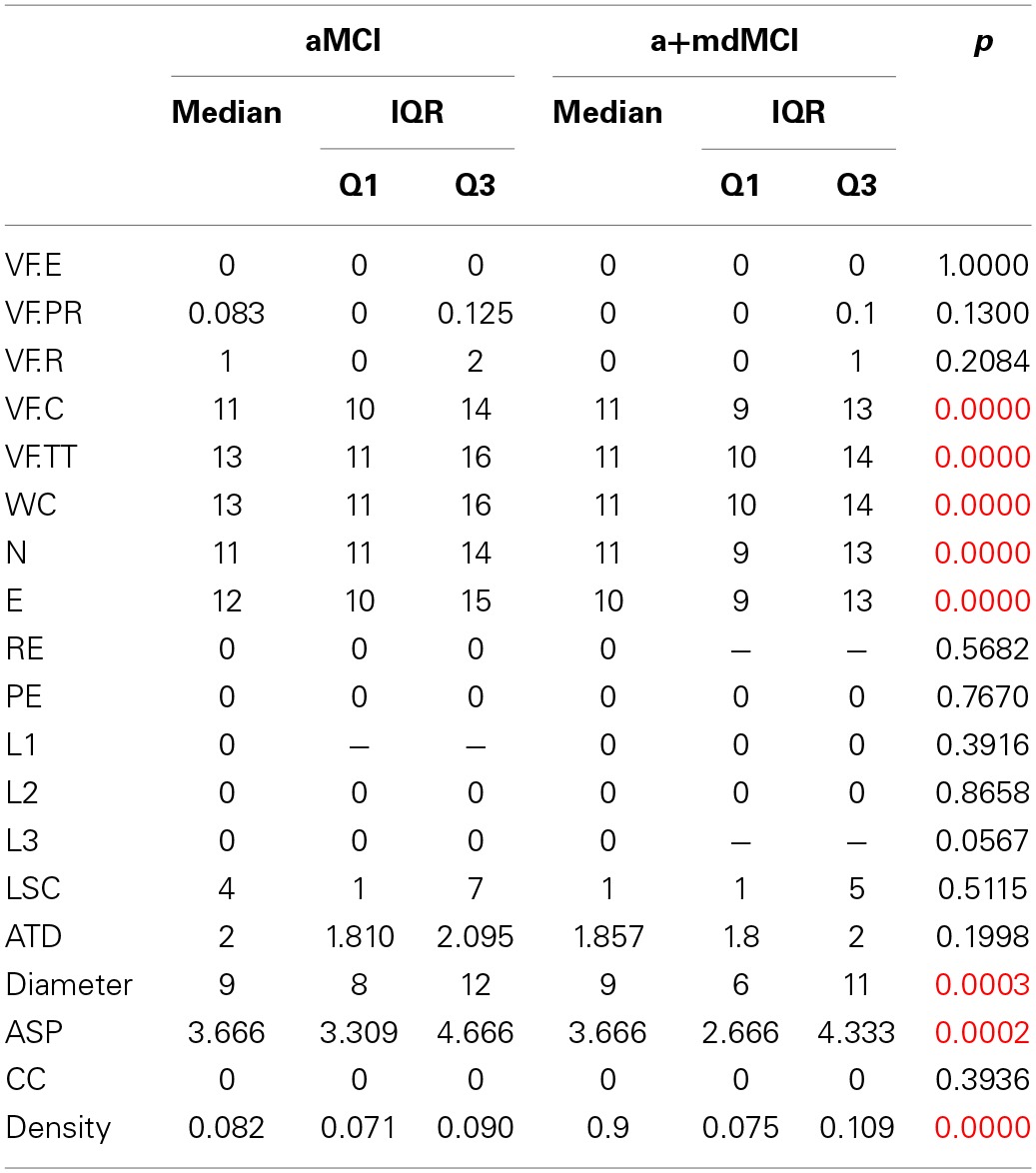
**Additional description of verbal fluency and Speech Graph Attributes for the MCI subtypes, and the four groups comparison**.

*Red values have significance p = 0.0083*.

A comparison of the four groups showed significant differences in daily functionality, general cognitive status, total and correct words produced, and in the SGA word count, nodes, edges, diameter, ASP and density (same attributes found in the three-group comparison).

**Figure 3:**
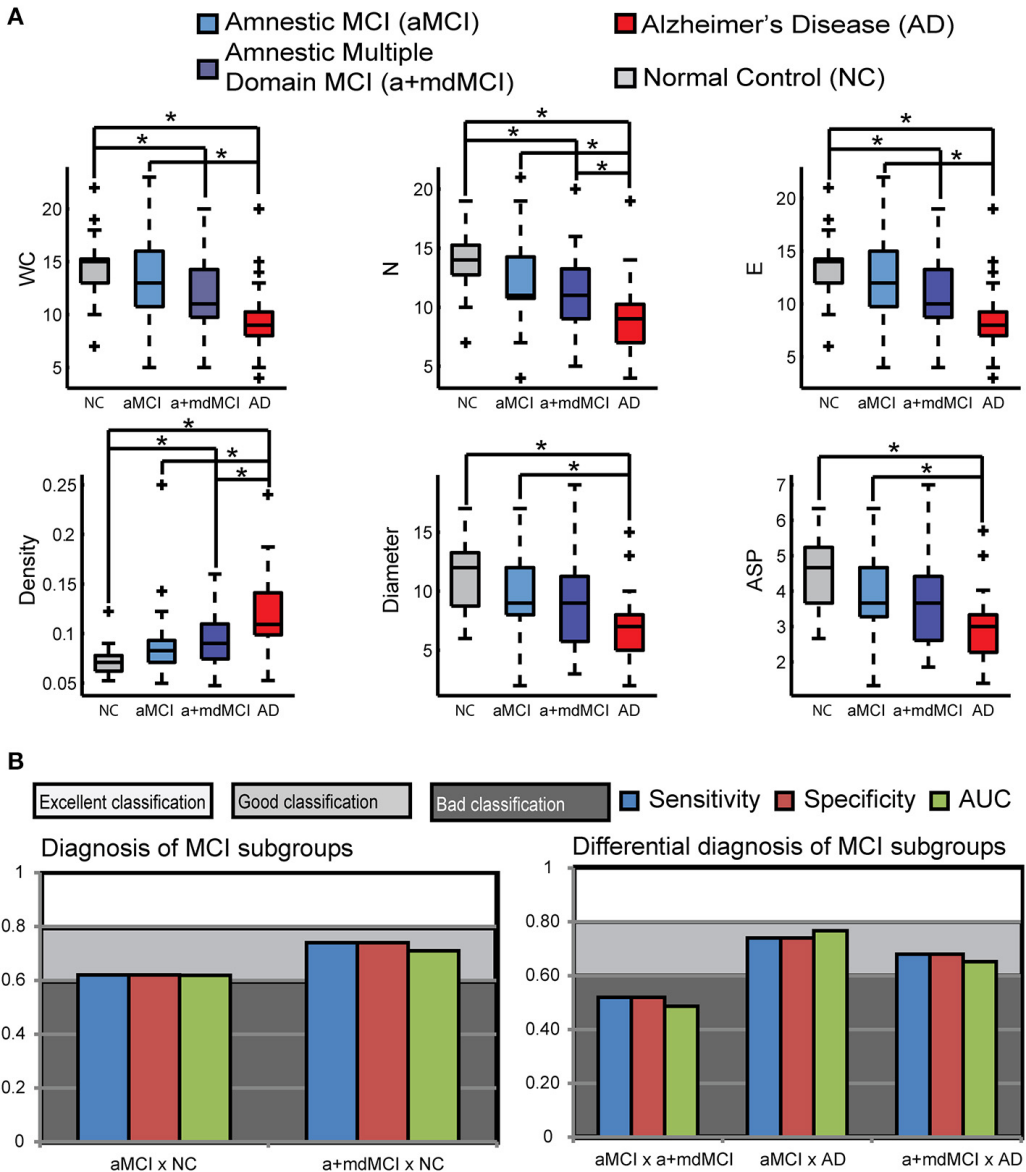
**Speech Graph Attributes (SGA) differentiates psychopathological MCI subgroups**. **(A)** SGA boxplots with significant differences among Alzheimer Disorder (AD), Amnesic Moderate Cognitive Impairment (aMCI), Multiple Domain Moderate Cognitive Impairment (a+mdMCI), and control groups indicated (*N* = 25 per group; Kruskal-Wallis test followed by two-sided Wilcoxon Rank-sum test with Bonferroni correction with alpha = 0.0083). **(B)** Rating quality measured by AUC, sensitivity and specificity, using SGA correlated with clinical symptoms measured with MMSE and Lawton scales (Table 4) (attributes: WC, N, E, Density, Diameter, and ASP). Notice that it is possible to sort the MCI subgroups from the NC or AD groups, but not one from another. Classification quality was considered excellent when AUC was higher than 0.8, good when AUC ranged from 0.6 to 0.8, and poor (not above the chance), when AUC was smaller than 0.6. ^*^ *p* = 0.0083.

Table 8 and Figure 3A compare the four groups of elderly, with Bonferroni correction for multiple comparisons (alpha = 0.0083).

**Table 8:**
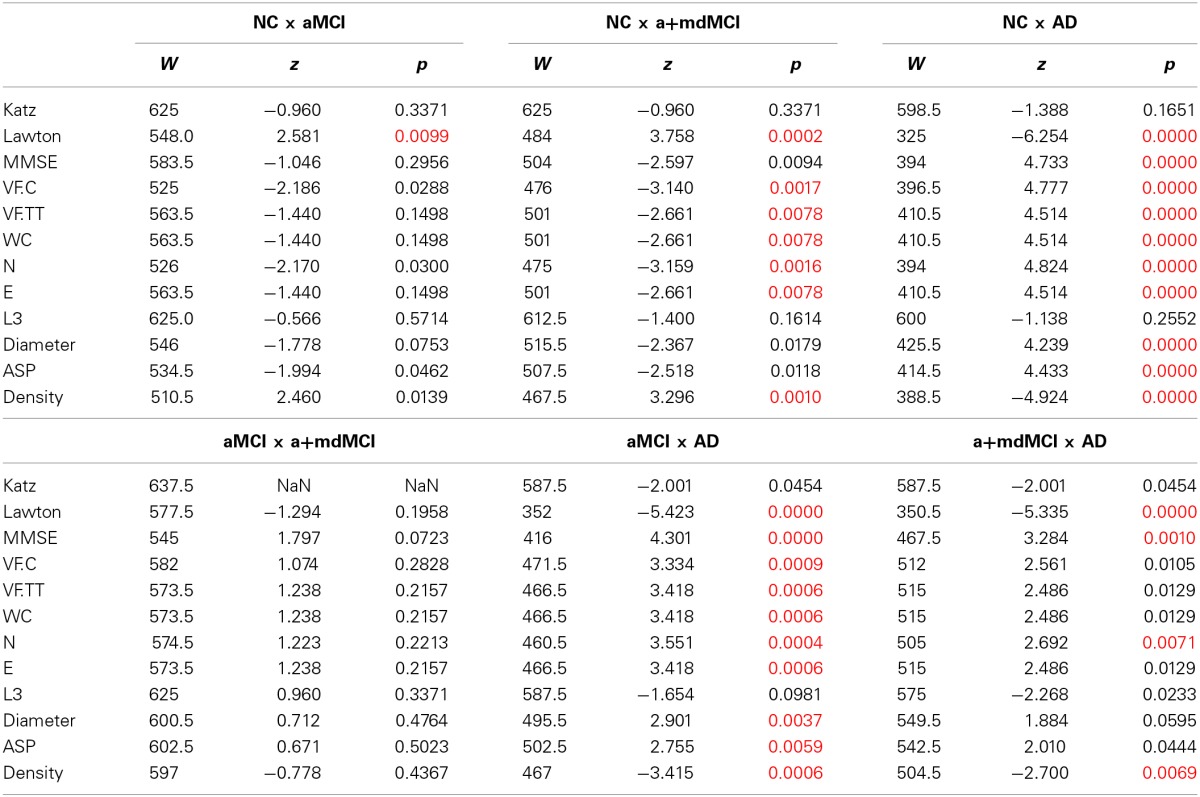
**Pairwise group comparison in the variables with significant difference across the four groups**.

*Red values have significance p = 0.0083*.

The pairwise comparison detected no significant differences between MCI subtypes in the measures selected in this study. The difference between the NC and aMCI groups occurred only in instrumental daily living functionality, i.e., NC are more independent than aMCI. The significant differences between the NC and AD and between aMCI and AD are similar; the NC and aMCI groups are less functionally dependent, have better cognitive status, produce more total and correct words, a higher word count, more nodes and edges, higher Diameter and ASP, and less dense networks when compared to the AD group. The NC are more functionally independent, produce more total and correct words, a higher word count, more nodes and edges, and a network less dense than the a+mdMCI group. AD subjects, comparable to the a+mdMCI group, were more functionally dependent, showed general cognitive impairment, and produced fewer nodes and a denser network.

The Naïve Bayes classifier results (Figure 3B) indicate that the selected SGA has a good classification power to the diagnosis of MCI subtypes against cognitive healthy aging, and also a good classification against the dementia group. On the other hand, SGA yielded a poor classification when used to distinguish between the two subtypes of MCI. When SGA were combined with the Lawton Index, we observed an increase in the power of classification across the four groups, except between the two MCI subtypes.

The combination of the SGA with the MMSE, showed less power when compared to the combination with the Lawton index; the combination of these three variables barely improved the classification beyond the SGA and Lawton combination. These results indicate that the combination of graph measures and functional dependence again provides for good classification across the three groups (AUC = 0.71–0.85), except between the MCI subtypes (AUC = 0.47).

## Discussion

The aim of the present study was to assess graph-theoretical differences in the execution of a verbal fluency task among elderly with normal and pathological aging. Our results demonstrate that SGA differed significantly among the AD, MCI, and NC groups and it could be used to classify the groups. The present results show the potential of graph analysis of verbal fluency task to discriminate between these groups in clinical practice.

The correlation between the SGA and the MMSE or the Lawton Index indicate that the SGA are associated with the general cognitive status and functional performance, two important clinical measures used in geriatric assessment. Patients with worse scores in the MMSE produced fewer numbers of nodes and a less dense network. As the functional performance decreases, indicating more severe cognitive impairment stages, the networks became denser, with a smaller diameter and average short path and with fewer numbers of nodes and edges. Their networks became smaller in the number of words, with a small path through the first word to the last one, and their animals have more connection with different neighbors than would be necessary. Subjects more cognitively impaired tended to perform more dependently on their daily activities. Importantly, some attributes of SGA could indicate the progression of cognitive impairment and functional decline, as shown by denser and smaller networks, with a fewer number of nodes, in subjects with more severe cognitive impairment.

Application of speech graph analysis for sorting the groups showed moderate to good classification quality. When selected SGA were combined to the Lawton Index, better classification were obtained, suggesting that the combination of these two simple tools of network measure and functionality can provide to the clinician a good indication of differential diagnosis, except for the contrast between the two MCI subtypes, which spanned a continuum and did not allow the differentiation and classification of the two groups.

The differences prevalent across all groups were in the global attributes of diameter, density and average shortest path (ASP). The results indicate that the networks built by the normal control elderly were more direct, without reoccurrence of words, resulting in a less dense network. Conversely, cognitive impairment corresponded to denser and less direct networks. The density differences across the groups were, among all comparisons, the most uniform result, except for the comparison between the two MCI subgroups, which yielded a pattern of continuous performance. The progressive worsening of cognitive performance within the MCI subtypes is consistent in the literature, indicating that a group of subtle deficits underlie the differential diagnosis (Diniz et al., 2007; Radanovic et al., 2009).

Even the groups that did not differ in total number of word repetitions differ in the occurrence of loops of 3 nodes (L3). Nearly all subjects, as expected, managed to avoid recurrences, but 20% of the AD subjects repeated the same word with only two words of interval (e.g., dog-cat-horse-dog). According to Huntley and Howard (2010), subjects with AD already have working memory deficits at the earliest stages of the disease. The impairment in central executive and episodic buffer functions of working memory probably stems from the difficulty of keeping information in mind while keeping the search for new information. These deficits probably explain the repetition of words in verbal fluency tasks with a very small interval.

The results outline a field that needs to be further explored in future studies, involving the density of the networks and the strength between the words in the semantic memory of elderly with pathological aging. The Parallel Distributed Processing Approach of Semantic Cognition predicts that the decrease in strength of the links between words in a semantic network may allow connections between pairs of words that would not be preferential under normal circumstances (McClelland and Rogers, 2003). Another aspect that deserves further investigation is the absence of difference across the groups in the connectivity attributes (LSC, ATD, and CC). This raises the hypothesis that even very different networks can share a similar structure of local connections, in which a small portion of the words are highly connected with other less connected words, maintaining the integrity of the network's general connection (Bales and Johnson, 2006; De Deyne and Storms, 2008).

Considering the graph analysis performed in this study, build-up in a co-occurrence of the words and based on the temporal link between them, future studies should consider multidimensional scaling and hierarchical clustering analysis. These types of analyses will represent the relation between the variables and combine it into groups, enhancing the results. Future studies should also address the differences between MCI patients and other neurological conditions in which cognitive impairments are quite similar, for example, Temporal Lobe Epilepsy (Holler and Trinka, 2014), as well as the potential association between graph analysis, neuroimaging and other diagnosis instruments. Furthermore, longitudinal studies are also necessary to evaluate whether SGA can help to identify MCI subjects with higher risk of progressing to Alzheimer's disease. In conclusion, the results suggest that SGA may be a useful tool to help in the differential diagnosis between MCI and AD.

### Conflict of interest statement

The authors declare that the research was conducted in the absence of any commercial or financial relationships that could be construed as a potential conflict of interest.
