# Supplementary material for: Graph analysis of verbal fluency test discriminate between patients with Alzheimer's disease, mild cognitive impairment and normal elderly controls
Source: Front Aging Neurosci. 2014 Jul 29;6:185. doi: 10.3389/fnagi.2014.00185 (PMC4114204; doi:10.3389/fnagi.2014.00185)
Supplement: Supplementary file 1 [file DataSheet1.DOCX]

Supplementary Table: Speech Graph Attributes

| **N:** Number of nodes. |
| --- |
| **E:** Number of edges. |
| **RE (Repeated Edges):** sum of all edges linking the same pair of nodes. |
| **PE (Parallel Edges):** sum of all parallel edges linking the same pair of nodes given that the source node of an edge could be the target node of the parallel edge. |
| **L1 (Loop of one node):** sum of all edges linking a node with itself, calculated as the trace of the adjacency matrix. |
| **L2 (Loop of two nodes):** sum of all loops containing two nodes, calculated by the trace of the squared adjacency matrix divided by two. |
| **L3 (Loop of three nodes):** sum of all loops containing three nodes (triangles), calculated by the trace of the cubed adjacency matrix divided by three. |
| **LSC (Largest Strongly Connected Component):** number of nodes in the maximal subgraph in which all pairs of nodes are reachable from one another in the directed subgraph (node a reaches node b, and b reaches a). |
| **ATD (Average Total Degree):** given a node n, the Total Degree is the sum of “in and out” edges. Average Total Degree is the sum of Total Degree of all nodes divided by the number of nodes. |
| **Diameter:** length of the longest shortest path between the node pairs of a network. |
| **Average Shortest Path (ASP):** average length of the shortest path between pairs of nodes of a network. |
| **Average Clustering Coefficient (CC):** Given a node n, the Clustering Coefficient Map (CCMap) is the set of fractions of all n neighbours that are also neighbours of each other. Average CC is the sum of the Clustering Coefficients of all nodes in the CCMap divided by number of elements in the CCMap. |
| **Density:** number of edges divided by possible edges. (D = E/N^2^), where E is the number of edges and N is the number of nodes. |
