# Supplementary material for: Graph analysis of verbal fluency test discriminate between patients with Alzheimer's disease, mild cognitive impairment and normal elderly controls
Source: Front Aging Neurosci. 2014 Jul 29;6:185. doi: 10.3389/fnagi.2014.00185 (PMC4114204; doi:10.3389/fnagi.2014.00185)
Supplement: Supplementary file 2 [file DataSheet2.PDF]

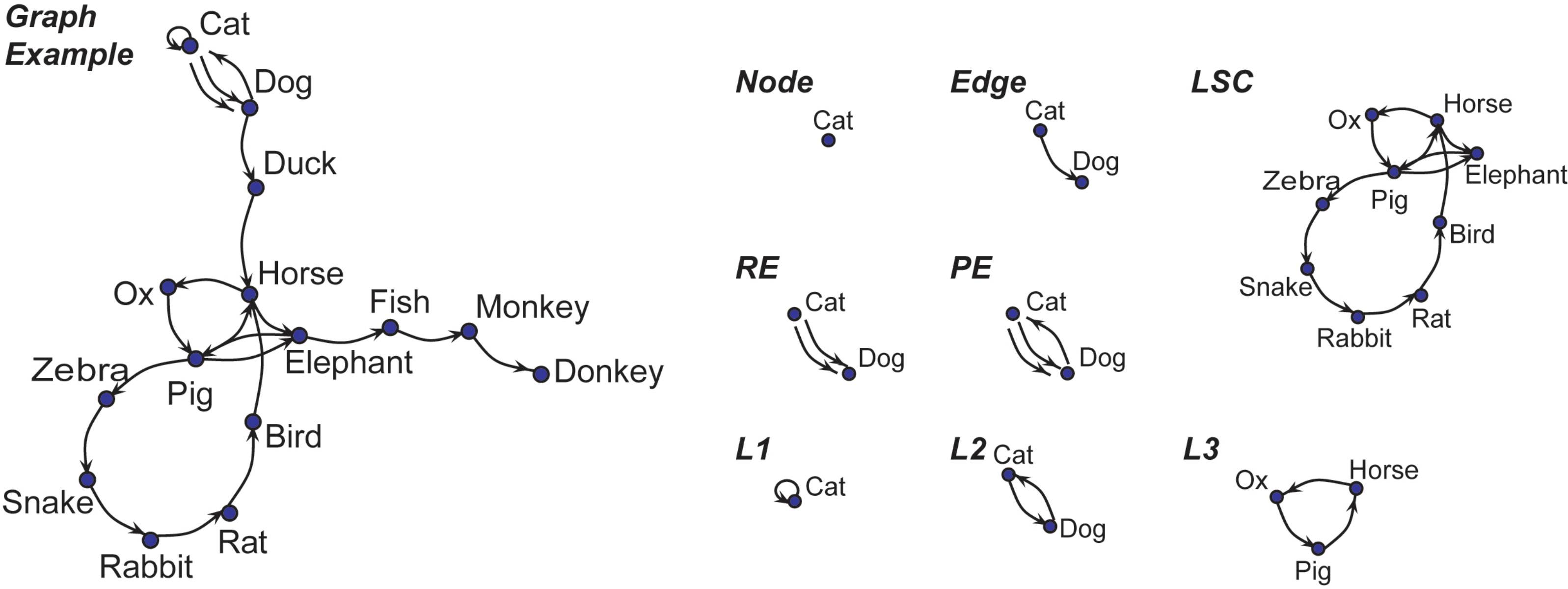

Supplementary Figure: Graph example to illustrate Speech Graph Attributes (SGA). On this graph we have 15 Nodes (words), 21 Edges (temporal links representing word sequence), 3 PE (parallel edge), 2 RE (repeated edge), 1 loops of one (L1), two (L2) and three (L3) nodes and the largest strongly connected component (LSC) is 9 (there is 9 nodes on the LSC).
